# Plasticity of coral physiology under ocean acidification

**DOI:** 10.18632/oncotarget.4983

**Published:** 2015-07-23

**Authors:** Alexander Ashley Venn, Eric Tambutté, Sylvie Tambutté

**Affiliations:** Marine Biology Department, Centre Scientifique de Monaco, Monaco, Monaco and Laboratoire International Associé 647 « BIOSENSIB », Centre Scientifique de Monaco-Centre National de la Recherche Scientifique, Monaco

**Keywords:** Chromosome Section, calcification, ion regulation, climate change, skeleton

Coral reefs are oases of life in the oceans, harbouring more than a quarter of all marine species. These vibrant ecosystems are founded on reef structures that are built by the CaCO_3_ skeletons of “stony” scleractinian corals. While productive and biodiverse, coral reef ecosystems are sensitive to many elements of global environmental change, including “ocean acidification” which impairs the capacity of corals to build their skeletons by calcification [1]. Ocean acidification is driven by seawater-uptake of rising atmospheric pCO_2_ and involves changes in seawater carbonate chemistry associated with decreasing seawater pH. Meta-analysis of published data suggests that ocean acidification will cause a worrying decline in reef coral calcification rates by the end of the 21^st^ century [2], but exactly how and why ocean acidification affects coral calcification isn't well understood at a physiological level.

**Figure 1:**
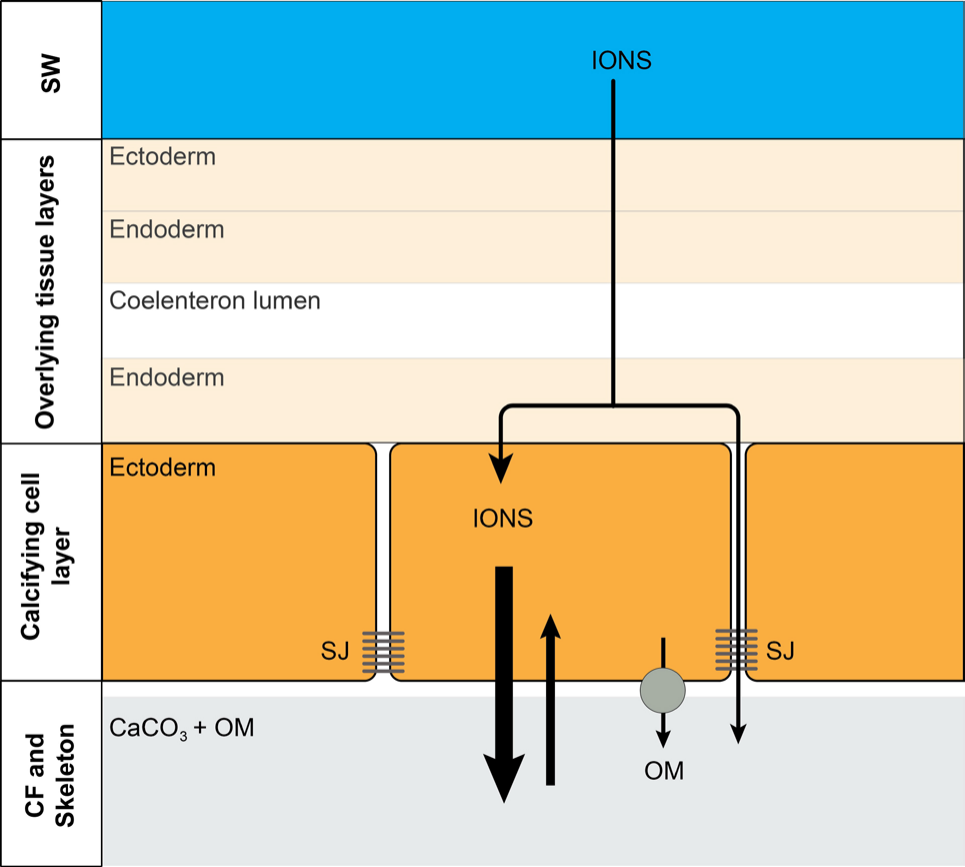
Schematic representation of coral histology and processes involved in calcification in corals Compartments are not represented according to their relative sizes. SW= seawater; CF = calcifying fluid; OM = organic matrix; SJ= septate junction.

Improving our understanding of the effects of ocean acidification on corals hinges on achieving a mechanistic understanding of the coral calcification process, yet relatively few cellular-level studies have addressed this topic [3]. Corals are diploblastic animals, characterised by a relatively thin layer of tissue overlying a complex CaCO_3_ skeleton. The calcifying cell layer that deposits the skeleton regulates ion supply to an internal calcifying fluid where the skeleton forms, and also produces an organic matrix which is incorporated into the skeleton's structure (Figure 1). Ions used for calcification arrive in the calcifying fluid *via* a paracellular pathway characterised by septate junctions, and *via* transcellular pathways involving ion transporters and channels in the calcifying cells. Certain of these ion transporters act to regulate conditions in the calcifying fluid, notably by elevating pH and boosting dissolved inorganic carbon (DIC) concentrations, to promote the precipitation of CaCO_3_ [4].

To better understand how ocean acidification affects coral calcification, we need to look for clues in both the coral tissues and the skeleton itself. In our recent work, published in Nature Communications [5], we carried out a controlled laboratory study on a model stony coral species, *Stylophora pistillata*, which can tolerate long-term exposure to low seawater pH. After more than a year at a low seawater pH that should favour dissolution of CaCO_3_, our corals continued to calcify, albeit at slower rates than controls. Interestingly however, the coral skeletons that formed at lower pH were significantly more porous and less dense than their counterparts at normal seawater pH. Our analyses ruled-out the possibility that physicochemical dissolution of the skeleton by low seawater pH had caused the observed increases in porosity. Indeed, pH measurements by confocal microscopy showed that the calcifying cell layer regulated calcifying fluid pH to elevated values that should favour CaCO_3_ precipitation, not dissolution. Rather, the explanation underlying why coral skeletons became more porous lay in a biologically-controlled shift in the coral skeleton's architecture, pointing to morphological plasticity of *S. pistillata* under seawater acidification.

These findings raise numerous questions about the molecular and cellular mechanisms controlling calcification, and we are currently employing genomic, transcriptomic and post-genomic approaches to get insight into the cellular physiology of corals under ocean acidification. For example how do corals control the chemistry of the calcifying fluid when the pH and carbonate chemistry of the surrounding seawater changes? Are genes coding for transporters involved in pH regulation responsive to acidification and/or do we see changes in transporter activity? Furthermore, separation of the calcifying fluid from the external seawater relies on septate junctions between cells that determine the permeability of the tissues. Do we see differential expression of genes coding for junctions and/or changes in functional properties of these junctions at different levels of seawater pH?

Some of the most intriguing questions pertain to what controls the morphological plasticity of the coral skeleton under ocean acidification. Interestingly, we observed that the change in morphology was accompanied by an increase in organic matrix protein incorporation into the skeleton. We know that certain organic matrix proteins can catalyse precipitation of CaCO_3_ [6], thus an increase in organic matrix protein secretion by the calcifying cells might occur in order to facilitate calcification at lower seawater pH, but could certain organic matrix proteins also play a role in controlling skeletal morphology? Organic matrix proteins function in this way in certain other calcifying systems [7], and this possibility is an exciting avenue of research for corals. In any case, we have much to learn about the fundamentals of coral calcification and its response to ocean acidification. Only through more physiological research at a cellular mechanistic level will we begin to decipher how corals produce complex and often ornate skeletons that underlie some of the most biodiverse, yet environmentally sensitive habitats on the planet.
